# Time-dependent changes and potential mechanisms of glucose-lipid metabolic disorders associated with chronic clozapine or olanzapine treatment in rats

**DOI:** 10.1038/s41598-017-02884-w

**Published:** 2017-06-05

**Authors:** Xuemei Liu, Zhixiang Wu, Jiamei Lian, Chang-Hua Hu, Xu-Feng Huang, Chao Deng

**Affiliations:** 1 School of Pharmaceutical Sciences, Southwest University, Chongqing, 400715 PR China; 2 Illawarra Health and Medical Research Institute, Wollongong, 2522 NSW Australia; 3 School of Medicine, University of Wollongong, Wollongong, 2522 NSW Australia

## Abstract

Chronic treatment with second-generation antipsychotic drugs (SGAs) has been associated with an increased risk of metabolic syndrome. To evaluate the longitudinal changes in glucose-lipid homeostasis after SGA use, we studied the time-dependent effects of olanzapine (OLZ) (3 mg/kg, b.i.d.) or clozapine (CLZ) (20 mg/kg, b.i.d.) treatment on metabolic profiles for 9 weeks in rats. Although only OLZ significantly increased body weight in rats, both OLZ and CLZ elevated blood lipid levels. Chronic OLZ treatment induced significant weight gain leading to a higher fasting insulin level and impaired glucose tolerance, whereas CLZ lowered fasting insulin levels and impaired glucose tolerance independent of weight gain. Treatment with both drugs deranged AKT/GSK phosphorylation and up-regulated muscarinic M3 receptors in the rats’ livers. Consistent with an elevation in lipid levels, both OLZ and CLZ significantly increased the protein levels of nuclear sterol regulatory element-binding proteins (SREBPs) in the liver, which was associated with improvement in hepatic histamine H1R. However, enhanced carbohydrate response element binding protein (ChREBP) signalling was observed in only CLZ-treated rats. These results suggest that SGA-induced glucose-lipid metabolic disturbances could be independent of weight gain, possibly through activation of SREBP/ChREBP in the liver.

## Introduction

Second-generation antipsychotic drugs (SGAs) are widely used in the treatment of schizophrenia, bipolar and other mental disorders^1, 2^. Numerous reports have linked SGAs, especially CLZ and OLZ, to metabolic disorders including weight gain, obesity, diabetes mellitus, and dyslipidemia^3–6^. The mechanisms of SGA-induced metabolic disturbance are likely to be multi-factorial and to involve both peripheral and central mechanisms^7–10^. Weight gain, resulting partly from increased appetite stimulation via blockade of hypothalamic 5HT_2C_ and H1 receptors, is a common side effect induced by SGAs^11, 12^. The other metabolic disturbances could be partly a consequence of weight gain caused by SGA treatment^13, 14^. However, recent evidence suggested that SGAs could directly elevate fasting triglyceride levels and increase insulin resistance without changes in body weight^15, 16^, and that antipsychotic treatment could cause impaired glucose regulation independent of adiposity, as non-obese patients on CLZ and OLZ still displayed significant insulin resistance^17, 18^. Although both of these SGAs caused the most weight gain in humans, it was interesting that unlike OLZ, CLZ did not cause significant weight gain in rodents, even though it induced other metabolic disorders^19–21^. Comparing the effects of OLZ and CLZ provides an excellent opportunity to investigate the role of weight gain in metabolic disorders caused by SGAs.

Dyslipidemia and insulin resistance could be the earliest detectable metabolic abnormalities in clinical patients treated with SGAs, which could eventually proceed to prediabetes, pancreatic β-cell failure, and then type II diabetes^16^. Chronic treatment, or even a single dose of CLZ or OLZ, could induce elevation of serum free fatty acids, followed by hepatic accumulation of lipids^22, 23^. These lipid changes are known to cause peripheral insulin resistance by inhibiting insulin-stimulated glucose uptake^24^. Insulin resistance was commonly associated with compensatory hyperinsulinemia, which also contributed to the pathogenesis of dyslipidemia. Previous evidence has shown that the abnormal lipid and cholesterol synthesis causedby CLZ or OLZ were associated with up-regulation of hepatic sterol regulatory element-binding proteins (SREBPs, including SREBP-1c and SREBP-2) and their associated target genes^25–29^. Notably, the expression of hepatic lipogenic genes was enhanced in insulin resistance^27, 30^, however the underlying mechanism was unclear. The carbohydrate response element binding protein (ChREBP) was a key player in the induction of genes of *de novo* fatty acid synthesis (lipogenesis) in response to glucose. Recent studies have shown that an active lipogenesis via ChREBP activation was associated with improved insulin sensitivity in adipose tissue and in the liver in mice^31–33^. It was important to evaluate the longitudinal change in glucose-lipid homeostasis and to further explore the potential mechanism in metabolic disturbance associated with SGA use.

Accumulated evidence has shown a potential relationship between antipsychotic drug affinity for specific receptors and metabolic side effects. Histamine H1 receptor (H_1_R)^34, 35^ might be responsible for SGA-induced weight gain, while muscarinic M3 receptors (M_3_R) were responsible for the gluco-metabolic side effects of SGAs^36, 37^. These receptors are widely distributed throughout the body, playing an important role in the normal maintenance of energy and glucose homeostasis, including regulation of hepatic, gastroentero, and pancreatic functions^38, 39^. Recently, co-treatment with betahistine (a histamine H1 receptor agonist) has been shown to improve OLZ-induced dyslipidemia by acting on the hepatic H_1_R^35^. Therefore, it was important to investigate how histaminergic and muscarinic receptors mediate metabolic disorders in chronic SGA-treated animal models. In addition, to gain insight into the time-dependent effects of SGAs on glucose and lipid homeostasis, and to explore underlying mechanisms in SGA-induced metabolic disturbance, we assessed the time-dependent effects of OLZ/CLZ treatment on lipid and gluco-metabolic function over time for 9 weeks, and further investigated the effects of chronic SGA treatment on the hepatic SREBPs/ChREBP pathways, muscarinic M_3_R, and histamine H_1_R in rats.

## Results

### Effects of chronic drug treatment on weight gain, food intake, white adipose tissue, liver and feeding efficiency

**Figure 1:**
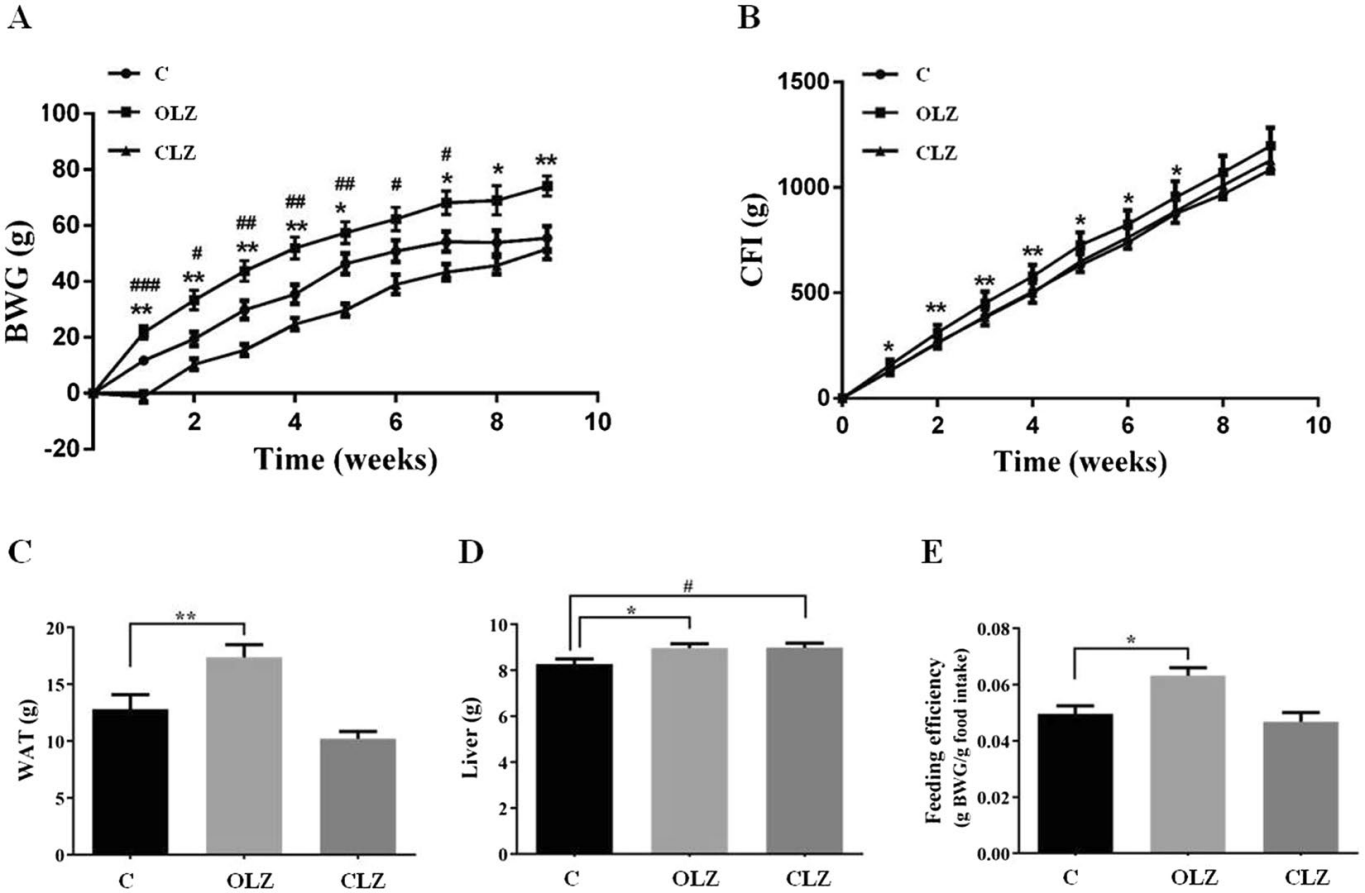
The effects of OLZ and CLZ treatment on (**A**) body mass, (**B**) food intake, (**C**) WAT, (**D**) liver and (**E**) feeding efficiency (gram weight gain/gram food intake). Rats were administrated orally with OLZ (3 mg/kg, b.i.d), CLZ (20 mg/kg, b.i.d) or vehicle for 9 weeks. Data are presented as mean ± SEM (n = 12 per group). *p < 0.05, **p < 0.01 and ***p < 0.001: OLZ treatment vs. control, ^#^ *p* < 0.05, ^##^ *p* < 0.01 and ^###^ *p* < 0.001: CLZ treatment vs. control. BWG: body weight gain; CFI: cumulative food intake; WAT: white adipose tissue, including perirenal, periovary, inguinal, and mesentery fat; C: control; OLZ: olanzapine; CLZ: clozapine.

Two-way repeated ANOVA (Treatment × Time as repeated measures) showed significant main effects of Weeks (F_9, 99_ = 306.4, *p* < 0.001), Treatment (F_2, 22_ = 20.82, *p* < 0.001), and a significant interaction between the two factors (F_18, 198_ = 6.145, *p* < 0.001) on accumulated body weight gain (BWG) (Fig. 1A). Post-hoc analysis identified a significant increase in BWG following OLZ treatment compared to the control throughout the treatment period (Week 1–5, *p* < 0.01; Week 7–9, *p* < 0.05), while a significant attenuation in weight gain was seen in the CLZ treatment group in Week 1–7 (*p* < 0.05). A one-way ANOVA revealed that there was a significant effect of Treatment on total cumulative food intake (CFI) (F_2, 22_ = 10.12, *p* < 0.001, Fig. 1B). OLZ induced a significant increase in CFI from Week 1–7 (Week 1, 5–7, *p* < 0.05; Week 2–4, *p* < 0.01, Fig. 1B), but not Week 8–9. However, no changes in CFI were observed in the CLZ-treated group, compared to the control (Fig. 1E). Notably, feeding efficiency (grams of weight gained/grams of food consumed) was significantly increased in animals treated with 1.0 mg/kg OLZ (*p* < 0.05), but not CLZ (*p* > 0.05), compared to the control (Fig. 1E).

At the endpoint of the experiment, a one-way ANOVA revealed a significant effect of Treatment on the total white adipose tissue (WAT) mass (the sum of the masses of perirenal, periovary, inguinal, and mesentery fat) (F_2,33_ = 11.84, *p* < 0.001, Fig. 1C). OLZ increased WAT compared with the controls (*p* < 0.01; Fig. 1C). The liver mass was recorded after chronic drug exposure, and the data revealed that both OLZ and CLZ treated rats had a larger liver than the controls (*p* < 0.05, Fig. 1D).

Progressive changes in blood and liver lipid composition, and effects on hepatic SREBP/ChREBP pathway following chronic drug treatment

**Figure 2:**
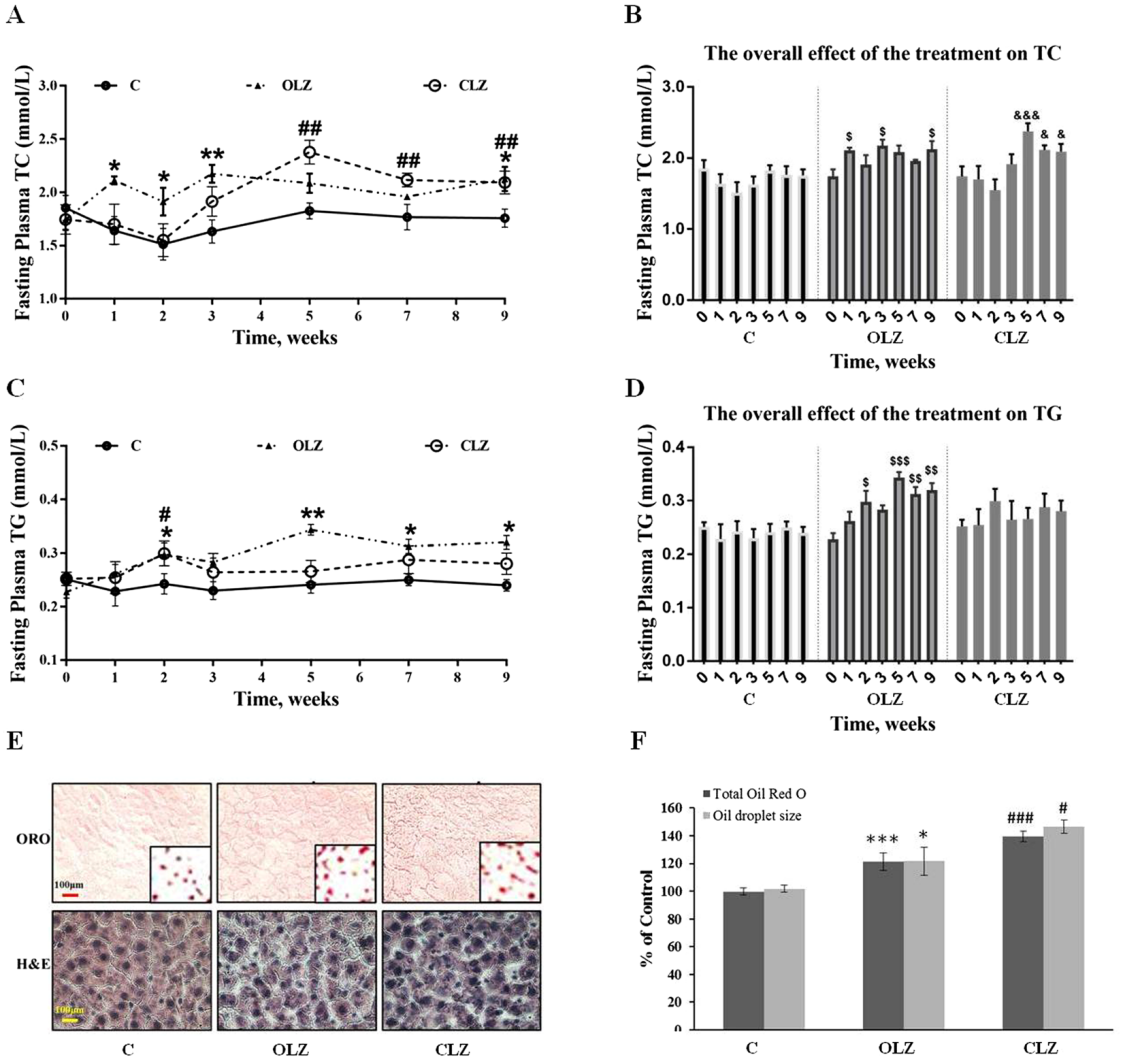
Chronic treatment of OLZ and CLZ led to progressive changes in blood and hepatic lipid composition. Time course of (**A**) fasting total cholesterol (TC) and (**C**) triglyceride (TG) in plasma during chronic drug treatment. The overall effect of the treatment on TC (**B**) and TG (**D**) using time as repeated measure and compared with the baseline on Week 0 of each group. (**E**) Oil Red O (ORO) and H&E staining of liver sections. Intense red colour indicates the presence of neutral lipids, mainly triglycerides. Scale bars, 100 µm. Inset images were magnified eight times in order to highlight the lipid-staining morphology. (**F**) Lipid level of liver. The data from ORO staining are presented. Data represent mean ± SEM (n = 12 per group). *p < 0.05, **p < 0.01, and ***p < 0.001: OLZ treatment vs. control, ^#^ *p* < 0.05, ^##^ *p* < 0.01, and ^###^ *p* < 0.001: CLZ treatment *vs*. control. ^$^ *p* < 0.05, ^$$^ *p* < 0.01, and ^$$$^ *p* < 0.001: OLZ treatment vs. baseline of OLZ group. ^&^ *p* < 0.05 and ^&&&^ *p* < 0.001: CLZ treatment vs. baseline of CLZ group. C: control; OLZ: olanzapine; CLZ: clozapine.

As shown in Fig. 2A,C, there was no difference in the baseline levels of total cholesterol (TC) and triglyceride (TG) between groups. A two-way repeated ANOVA (Treatment × Time as repeated measures) showed significant main effects of Treatment factor for fasting TC (F_2, 22_ = 6.972, *p* < 0.05) and TG levels (F_2, 33_ = 8.080, *p* < 0.01), as well as Time factor (F_6, 66_ = 5.805, *p* < 0.001; F_6, 66_ = 3.737, *p* < 0.05, respectively). There were also significant interactions between the two factors for TC levels (F_12,132_ = 3.056, *p* < 0.001) and TG levels (F_12, 198_ = 2.257, *p* < 0.05). Overall, OLZ treatment caused a fasting plasma TC increase throughout the treatment period (*p* = 0.018), and had a significantly higher TC level than the controlin Week 1–3 and Week 9 (all *p* < 0.05; Fig. 2A). Similarly, there was an overall increase in plasma TG levels (*p* = 0.024), particularly in Week 2 and Week 5–9 after OLZ treatment in comparison with the control (all *p* < 0.05; Fig. 2C). The overall effects of OLZ treatment on plasma TG and TC levels are also clear in comparison with its baseline (Week 0; Fig. 2B,D). On the other hand, CLZ treatment started to increase TC in Week 3, and became significant in Week 5–9 compared with both the control (all *p* < 0.01; Fig. 2A) and its baseline on Week 0 (Fig. 2B). CLZ also elevated TG from Week 2 compared with the control (*p* < 0.05; Fig. 2C).

Figure 2E showed representative images of randomly selected liver sections stained with Oil Red O (ORO) and H&E. As expected, ORO analysis showed that both the OLZ and CLZ treatment groups had significantly increased positive staining for neutral lipids (CLZ 139.50 ± 6.09 and OLZ 119.22 ± 4.01%, both *p* < 0.0001) and increased lipid droplet size (CLZ 146.59 ± 10.27 and OLZ 121.56 ± 4.88%, both *p* < 0.05) as indicated by ORO images compared with the control (Fig. 2E,F). The images counterstained with H&E showed that there were no notable histological changes in treatment groups compared with the control.

**Figure 3:**
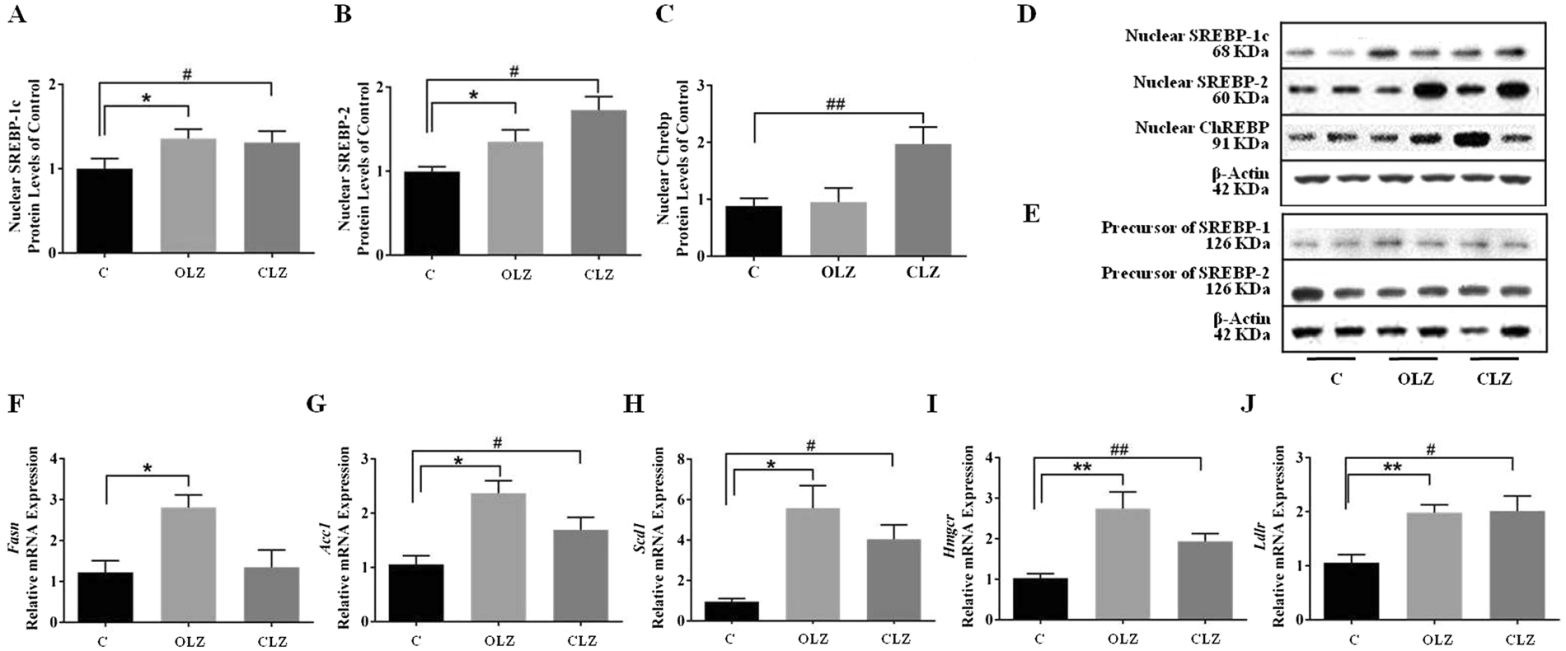
Effects of antipsychotic drugs on the SREBP and ChREBP pathway. Western blot analysis of the protein expression of hepatic (**A**) nuclear SREBP-1c, (**B**) nuclear SREBP-2, and (**C**) nuclear ChREBP protein after 9-week treatment of OLZ and CLZ. A representative blot is shown (**D** and **E**). The mRNA expression of their target genes was quantified and normalized to *β-actin* and *gapdh* by qPCR: *Fasn* (**F**), *Acc1* (**G**), *Scd1* (**H**), *Hmgcr* (**I**), and *Ldlr* (**J**). Data represent mean ± SEM of 2 independent experiments done in duplicate (n = 6 per group). *p < 0.05, **p < 0.01, and ***p < 0.001: OLZ treatment vs. control, ^#^ *p* < 0.05, ^##^ *p* < 0.01, and ^###^ *p* < 0.001: CLZ treatment vs. control. C: control; OLZ: olanzapine; CLZ: clozapine.

In order to demonstrate the involvement of the SREBPs/ChREBP pathway in mediating lipid metabolic disorders, we measured the levels of three transcriptional factors and quantified the transcriptional levels of their target genes. OLZ significantly augmented nuclear SREBP-1c and SREBP-2 protein levels (Fig. 3A,B,D, both *p* < 0.05), but not nuclear ChREBP (Fig. 3C,D, Supplementary information). Consistently, OLZ led to a significant increase in transcriptional activity in SREBP-1c target genes (*Fasn*, Fig. 3F, 2.8-fold, *p* < 0.05; *Acc1*, Fig. 3G, 2.4-fold, *p* < 0.05; and *Scd1*, Fig. 3H, 5.6-fold, *p* < 0.05), and in SREBP-2 target genes (*Hmgcr*, Fig. 3I, 2.7-fold, *p* < 0.01 and *Ldlr*, Fig. 3J, 2.0-fold, *p* < 0.01). CLZ also led to a marked increase in nuclear SREBP-1c (Fig. 3A,D, *p* < 0.05) and nuclear ChREBP levels (Fig. 3C,D, *p* < 0.01), which were associated with a significant up-regulation of *de novo* lipogenesis enzyme expression (*Acc1*, Fig. 3G, 1.7-fold, *p* < 0.05 and *Scd1*, Fig. 3H, 4.0-fold, *p* < 0.05). In particular, nuclear SREBP-2 (Fig. 3B,D, *p* < 0.05) and its target genes *(Hmgcr*, Fig. 3I, 1.9-fold, *p* < 0.01 and *Ldlr*, Fig. 3J, 2.0-fold, *p* < 0.05) were significantly increased. No significant change of the precursor of SREBPs was observed in the two drug treatment groups (Fig. 3E, Supplementary information).

### Progressive changes in Glucose Metabolism

**Figure 4:**
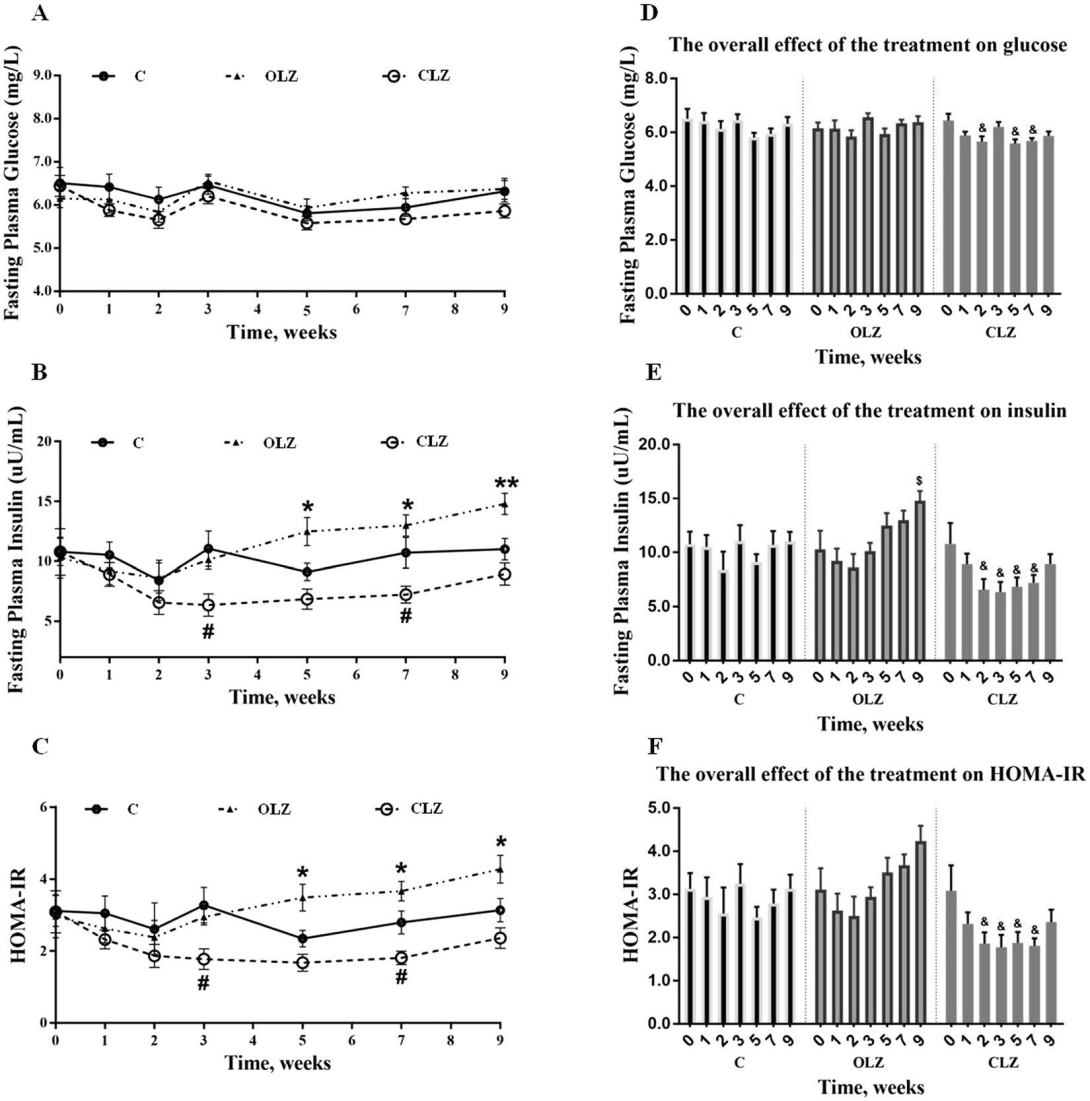
Chronic drug treatment led to progressive changes in blood glucose metabolism. Time course (mean ± SEM) of fasting plasma levels of glucose (**A**), insulin (**B**) and HOMA-IR (**C**) during chronic drug treatment. The overall effect of the treatment on glucose (**D**), insulin (**E**), and HOMA-IR (**F**) using time as repeated measure and compared with the baseline on Week 0 of each group. Animals received OLZ, CLZ, or cookie dough alone (control), as indicated. Data represent mean ± SEM (n = 12 per group). *p < 0.05 and **p < 0.01: OLZ treatment vs. control, ^#^ *p* < 0.05 and ^##^ *p* < 0.01: CLZ treatment *vs*. control. ^$^ *p* < 0.05, ^$$^ *p* < 0.01, and ^$$$^ *p* < 0.001: OLZ treatment vs. the baseline of OLZ group. ^&^ *p* < 0.05 and ^&&&^ *p* < 0.001: CLZ treatment vs. the baseline of CLZ group. C: control; O: OLZ; Cl: CLZ.

There were no significant differences between treatment groups in the baseline levels of glucose, insulin, and homeostasis model assessment insulin resistance index (HOMA-IR) (all *p* > 0.05). No significant changes in the levels of fasting glucose were detected at each time point between the OLZ or CLZ treatment group and the control group (Fig. 4A). However, a further overall comparison (using time as repeated measures) showed a statistically significant decrease in overall fasting glucose level at Weeks 2, 5 and 7 compared with the baseline (Week 0) in the CLZ group (*p* < 0.05, Fig. 4D). A two-way repeated ANOVA (Treatment × Time as repeated measures) on fasting insulin levels revealed significant effects of Treatment (F_2, 22_ = 8.923, *p* < 0.05) and Time (F_6, 66_ = 4.593, *p* < 0.001), but indicated no interaction between the two factors. After 5-week treatment, insulin levels showed a clear increase that reached significance at Weeks 5– 9 for the OLZ group compared with the control (all *p* < 0.05; Fig. 4B) or at Week 9 compared with the baseline (Week 0) of the OLZ group (Fig. 4E), following a continuous increase in HOMA-IR (to assess insulin resistance) which was consistent with insulin levels (Fig. 4C). On the contrary, chronic CLZ administration induced a decrease in plasma insulin levels and HOMA-IR throughout the treatment period (*p* = 0.042), particularly at Week 3 and 7 compared with the control group (both *p* < 0.05; Fig. 4B,C) or at Weeks 2–7 compared with the baseline (Week 0) of the CLZ group (all *p* < 0.05; Fig. 4E,F). Moreover, the insulin levels showed a tendency to be positively correlated with body weight change (Week 8, *r* = 0.428 and *P* = 0.098, and Week 9, *r* = 0.466 and *P* = 0.069) in the OLZ-treated group, suggesting that the changes in body weight gain might be partially associated with OLZ-induced hyperinsulinemia and insulin resistance, whereas CLZ treatment indicated a lack of this correlation.

### Immediate and chronic effects of SGAs on glucose metabolism

**Figure 5:**
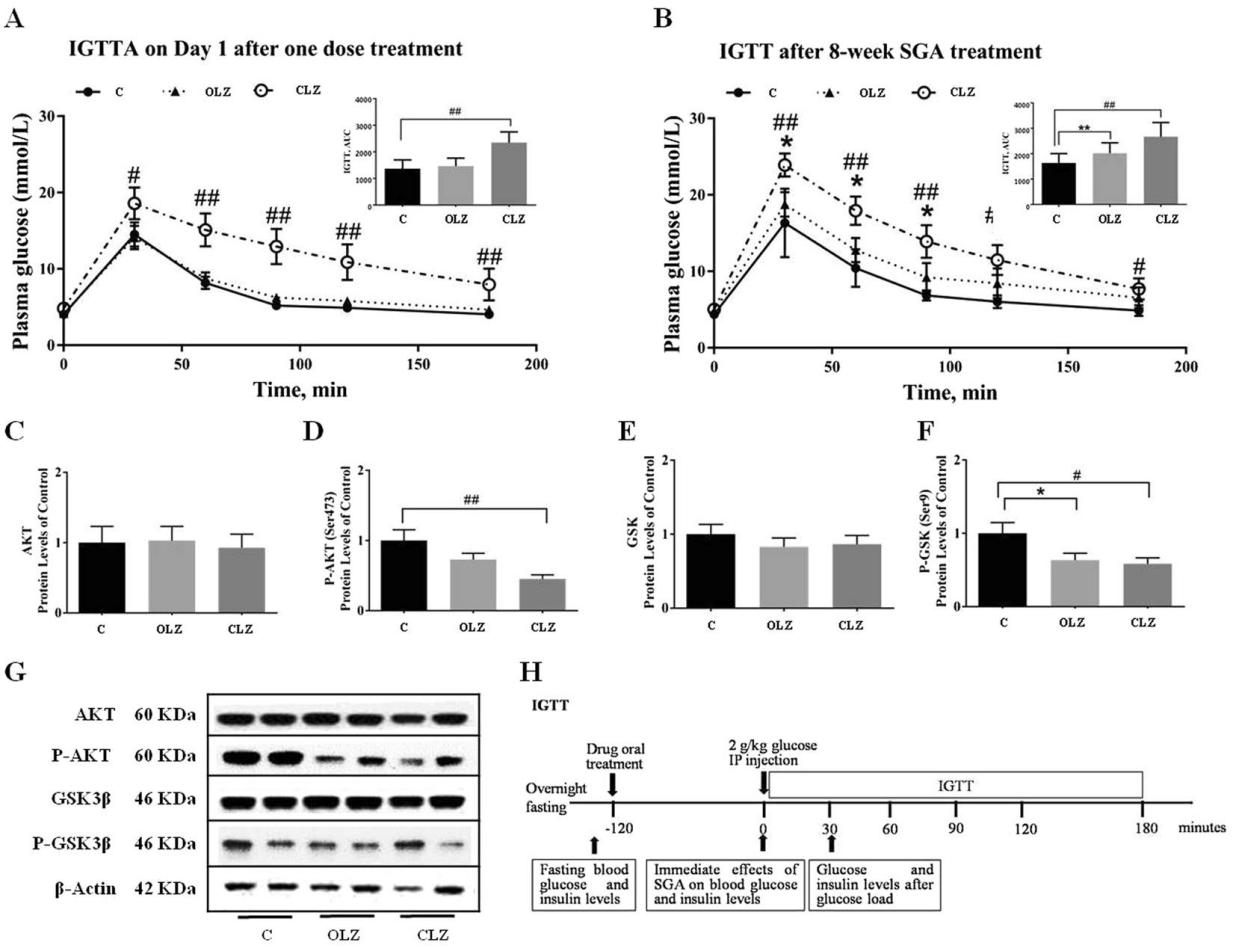
Effects of OLZ or CLZ on glucose metabolism. (**A** and **B**) IGTTs were conducted in rats after one dose treatment at Day 1 and after 8-week chronic OLZ or CLZ treatment. Data represent mean ± SEM (n = 12 per group). (**C–F**) Presents the levels of AKT, phosphor-AKT (Ser473), GSK, and phosphor-GSK (Ser9) in the liver. (**G**) Shows the representative western-blotting images. (**H**) Experimental protocol describing IGTT on Week 0 and 8. Data were normalised by taking the average value of the control group as 100% and expressed mean ± SEM of 2 independent experiments done in duplicate (n = 6 per group). *p < 0.05, **p < 0.01 and ***p < 0.001: OLZ treatment vs. control, ^#^ *p* < 0.05, ^##^ *p* < 0.01 and ^###^ *p* < 0.001: CLZ treatment vs. control. C: control; OLZ: olanzapine; CLZ: clozapine.

To investigate the immediate and chronic effects of OLZ and CLZ on glucose metabolism in rats, plasma insulin, glucose, and non-esterified fatty acid (NEFA) levels were determined following a single oral administration of 3 mg/kg OLZ or 20 mg/kg CLZ on Day 1 and after 8-week chronic OLZ or CLZ treatment. Although no significant effect of an oral single dose of OLZ or CLZ was observed in plasma glucose and insulin levels, during an Intraperitoneal Glucose Tolerance Test (IGTT), the glucose concentration over the 180 min and the areas under the glucose tolerance test curves (AUC) were significantly increased on Day 1 by the single dose CLZ treatment but not OLZ compared with the control group (Fig. 5A, *p* < 0.01). Moreover, the single dose OLZ or CLZ treatment had no effect on insulin concentration at 30 min following the post-glucose challenge (Table 1). Interestingly, after the 8-week drug treatment, although there was still no significant difference in plasma fasting glucose levels among the different groups, both OLZ and CLZ impaired glucose tolerance; the plasma glucose concentrations in the drug treatment groups were significantly higher than the control at several time points (Fig. 5B). Accordingly, AUC for plasma glucose during IGTTs following chronic drug treatment was significantly increased in both drug-treated groups compared with the control group (Fig. 5B, both *p* < 0.01). One-way ANOVA revealed the significant effects of Treatment on the plasma levels of insulin in the Week 8 experiment (F_2, 35_ = 8.135, *p* < 0.01). Compared with the controls, the plasma insulin levels were significantly higher than controls both 2 h after OLZ administration (*p* < 0.01) and 30 min after glucose administration (*p* < 0.05), whereas CLZ caused a significant decrease at 30 min in the post-glucose challenge (*p* < 0.05), as shown in Table 1. The insulin/glucose-ratio (I:G ratio) was consistent with the insulin change following the post-glucose challenge. Indeed, a markedly negative correlation between plasma glucose levels and insulin levels was observed in the OLZ-treated group at Week 8 (*r* = −0.390, *p* < 0.05). In contrast, CLZ treatment reduced the 30-min post-glucose challenge insulin levels, and further significantly lowered the I:G ratio (*p* < 0.01) (Table 1). Consistent with the promoted plasma insulin levels, the significantly increased levels of plasma NEFA were also observed in OLZ-treated rats in both single dose (*p* < 0.05) and 8-week(*p* < 0.01) treatment, but not in CLZ-treated rats, compared to controls (Table 1).

**Table 1:** Plasma glucose, insulin, and NEFA levels in response to one dose and 8-week drug treatment.

Characteristic		Control n = 12	OLZ n = 12	CLZ n = 12
Glucose				
Day 1 after one dosetreatment	Fasting levels, mmol/L	6.51 ± 1.26	6.15 ± 0.73	6.45 ± 0.84
	0 min postglucose-challenge, mmol/L (2 h after drug administration)	6.75 ± 1.28	6.92 ± 1.29	7.27 ± 0.79
	30 min postglucose-challenge, mmol/L	14.51 ± 5.45	14.11 ± 5.27	**18.58 ± 7.20** ^**#**^
	120 min postglucose-challenge, mmol/L	4.91 ± 1.02	5.82 ± 1.91	**10.87 ± 8.07** ^**##**^
	AUC	1241	1339	**2200** ^**#**^
After 8-week treatment	Fasting levels, mmol/L	6.32 ± 0.86	6.37 ± 0.81	5.86 ± 0.56
	0 min postglucose-challenge, mmol/L (2 h after drug administration)	6.34 ± 0.50	6.64 ± 0.89	6.40 ± 0.98
	30 min postglucose-challenge, mmol/L	16.34 ± 4.49	18.76 ± 5.53	**23.91 ± 5.18** ^**##**^
	120 min postglucose-challenge, mmol/L	6.03 ± 1.81	8.43 ± 6.76	**11.48 ± 6.81** ^**#**^
	AUC	1493	1872**	**2496** ^**##**^
Insulin				
Day 1 after one dose treatment	Fasting levels, uU/mL	10.78 ± 1.15	10.30 ± 1.70	10.79 ± 1.94
	0 min postglucose-challenge, uU/mL (2 h after drug administration)	11.39 ± 2.97	14.13 ± 4.99	13.29 ± 3.13
	30 min postglucose-challenge, uU/m	68.88 ± 23.20	66.03 ± 28.30	71.73 ± 27.44
	Insulin: Glucose Ratio (30 min)	4.74	4.68	3.86
	Fasting levels, uU/mL	11.01 ± 0.90	14.79 ± 0.90*	8.93 ± 0.92
After 8-week treatment	0 min postglucose-challenge, uU/mL (2 h after drug administration)	13.10 ± 1.95	19.29 ± 698**	10.63 ± 5.96
	30 min postglucose-challenge, uU/mL	72.25 ± 18.00	103.34 ± 30.26*	**49.92 ± 25.37** ^**#**^
	Insulin: Glucose Ratio (30 min)	4.42	5.51*	**2.09** ^**##**^
NEFA				
Day 1 after one dose treatment	Fasting levels, mmol/L	0.40 ± 0.18	0.39 ± 0.21	0.38 ± 0.15
	0 min postglucose-challenge, mmol/L (2 h after drug administration)	0.38 ± 0.10	0.51 ± 0.17*	0.46 ± 0.14
After 8-week treatment	Fasting levels, mmol/L	0.37 ± 0.15	0.37 ± 0.12	0.41 ± 0.09
	0 min postglucose-challenge, mmol/L (2 h after drug administration)	0.37 ± 0.06	0.46 ± 0.10**	0.39 ± 0.06

Data were obtained from 12 rats per group and represent mean ± SD. Biochemical determinations were made in duplicate (*p < 0.05 and **p < 0.01: OLZ treatment vs. control, ^#^ *p* < 0.05 and ^##^ *p* < 0.01: CLZ treatment vs. control).

To further address regulation of the peripheral insulin signalling pathway in chronic OLZ or CLZ treatment, we next evaluated AKT and GSK3β phosphorylation in the liver of these rats. As shown in Fig. 5D, OLZ tended to reduce pAKT (Ser 473) (*p* = 0.096) but CLZ significantly reduced its phosphorylation level (*p* < 0.01), which further significantly reduced GSK3β phosphorylation at Ser 9 (both *p* < 0.01; Fig. 5E,F). Consistent with chronic CLZ-induced insulin secretion deficiency, AKT and GSK3β phosphorylation levels were obviously reduced in rat livers compared to those in the control (both *p* < 0.05; Fig. 5D,F,G, Supplementary information). Total AKT and GSK3β protein levels were not altered by these treatments.

### Changes of M_3_R and H_1_R in rat liver in response to chronic treatment with OLZ or CLZ

**Figure 6:**
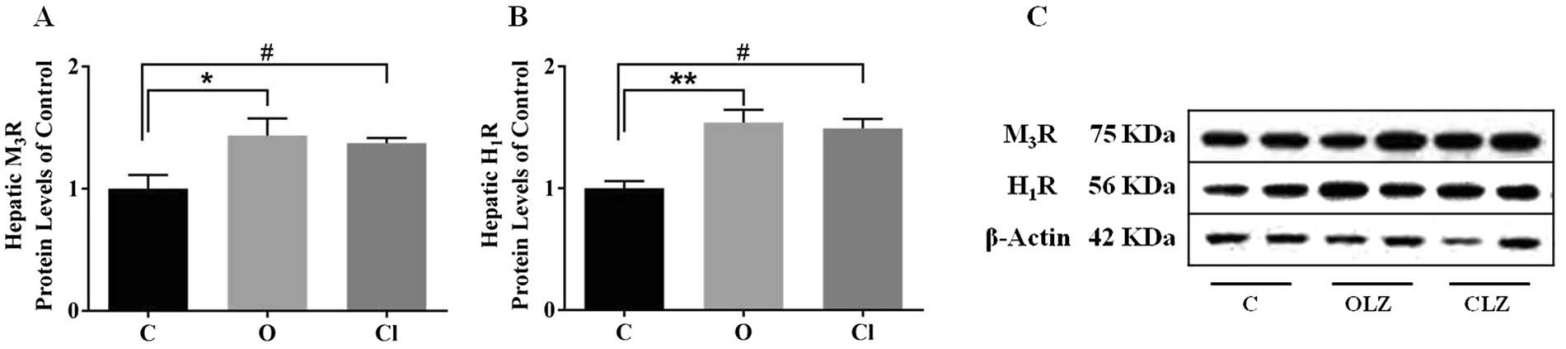
Response of hepatic M3-muscarinic receptors (M_3_R) and H1-histamine receptors (H_1_R) to chronic treatment with OLZ or CLZ. Western blot analysis of the protein expression of M_3_R (**A**) and H_1_R (**B**) (n = 6 per group) in liver tissue lysate. Representative blots are shown (**C**). All data are presented as mean ± SEM. *p < 0.05 and **p < 0.01: OLZ treatment vs. control, ^#^ *p* < 0.05: CLZ treatment *vs*. control. C: control; OLZ: olanzapine; CLZ: clozapine.

A one-way ANOVA revealed that drug Treatment had significant effects on the expression of H_1_R (F_2,17_ = 11.02, *p* = 0.006) and M_3_R (F_2,17_ = 9.252, *p* = 0.018) in the liver (Fig. 6, Supplementary information). Post-hoc comparisons further showed that significant effects were attributed to OLZ compared to the controls for H_1_R and M_3_R (both *p* < 0.05). Similarly, CLZ also induced a significant increase in the expression of these two hepatic receptors compared to controls (both *p* < 0.05). Our data also showed a significant positive correlation between hepatic H_1_R levels and nuclear SREBP-1 levels (*r* = 0.516, *p* = 0.049) as well as nuclear SREBP-2 levels (*r* = 0.691, *p* = 0.004), while H_1_R showed a tendency towards a significantly negative relationship with p-AKT (*r* = −0.574, *p* = 0.083) and p-GSK levels (*r* = −0.615, *p* = 0.058). Finally, it is worth noting that hepatic M_3_R levels were significantly correlated with nuclear SREBP-2 levels (*r* = 0.555, *p* = 0.032), but no significant correlation between M_3_R levels and p-AKT or p-GSK levels was found (*r* = −0.377, *p* = 0.166).

## Discussion

The present study evaluated the time-dependent effects of treatment with OLZ and CLZ at clinic equivalent doses on glucose-lipid homeostasis in adult female rats. Although only OLZ significantly increased body weight in rats, both OLZ and CLZ elevated blood lipid levels. During the treatment period, OLZ-treated rats had higher fasting insulin levels and HOMA-IR with mildly impaired glucose tolerance, whereas CLZ-treated rats showed significantly lowered fasting plasma insulin levels and impaired glucose tolerance compared with the control. Even then, deranged AKT/GSK phosphorylation and up-regulated hepatic muscarinic M_3_R were still observed in the two drug treatment groups. Consistent with an elevation in lipid levels, both OLZ and CLZ significantly increased the protein levels of nuclear SREBP-1c and SREBP-2in the liver, and led to a transcriptional up-regulation for their target genes, which was associated with up-regulation of hepatic histamine H_1_R. CLZ, but not OLZ, also mediated an increase in nuclear ChREBP, which further activated hepatic *de novo* lipogenesis. These results provide *in vivo* support for the hypothesis that the glucose and lipid metabolic disturbances caused by chronic treatment with SGAs could be, at least partly, independent of weight gain, possibly through activation of SREBP/ChREBP in the liver. Furthermore, acute and continuous impaired insulin secretion could contribute to metabolic syndrome under SGA therapy.

Previous studies on metabolic side-effects of SGA administration have measured the changes in glucose, lipid, and hormonal levels^40^. A 6-week treatment with antipsychotics was associated with significant increases in fasting glucose (mean change from baseline 0.36 mmol/L with OLZ, *p* < 0.01 for combined treatment group vs. control)^41^. OLZ and CLZ acutely impaired whole-body insulin sensitivity in a dose dependent manner (*p* < 0.001 vs. Vehicle)^42^. Wu and colleagues showed 8-week OLZ and CLZ treatment increased TC and TG in 112 treatment naïve in-patients^43^. However, there were some variable results, especially in lipid profiles in SGA-treated rats, which might reflect considerable differences in sex, dose, and duration of drug administration. As a result, in this study, the progressive changes of plasma glucose, lipid and insulin levels following OLZ and CLZ treatment were monitored from Week 1 to Week 9.

Even with no significant change in fasting glucose level, an increase in insulin level induced by chronic OLZ treatment was followed by a continuous increase in HOMA-IR which was consistent with insulin levels. On the contrary, chronic CLZ administration caused a decrease in plasma insulin levels and HOMA-IR throughout the treatment period, suggesting reduced insulin secretion. These alterations arising from CLZ treatment are not in accordance with a previous report that 10 mg/kg CLZ induce impaired glucose tolerance associated with increased insulin secretion in rats^44^. It could be due to the fact that a high dose (20 mg/kg) in this study might directly impair the secretion function of β-islets. Therefore, to better understand the mechanism of glucose metabolism regulation during treatment with SGAs, it is important in further studies to employ multiple doses to investigate the dose-response effects. To test the acute effect of CLZ and OLZ on glucose metabolism before any change in body composition, CLZ or OLZ was administered as a single dose on Day 1. In this case, we found that CLZ but not OLZ-treated rats presented with a significantly higher increase in plasma glucose levels under 2 g/kg glucose challenge compared to controls, even though both drugs had no significant effects on insulin release. These acute effects were adiposity independent, suggesting CLZ could have a direct effect on insulin sensitivity. After long-term drug treatment, still without significant weight gain, CLZ led to the further impairment of glucose-stimulated insulin secretion, and lower-activated AKT signalling, which was inline with the decreased insulin levels as shown in this study. Surprisingly, OLZ did not have significant acute effects on glucose metabolism, but may have a role in increasing insulin levels during chronic treatment. Even so, insulin signaling was not hyper-activated inline with the increased insulin levels in the liver. The increase in insulin levels during OLZ treatment may compensate for the increase in insulin resistance. It is worth noting that a further experiment with an acute dose of insulin may help us to understand the effects of insulin on AKT/GSK signaling. Lower-activated AKT signaling and the reduction of GSK3β phosphorylation caused by chronic treatment with SGAs might be associated with deranged insulin signaling^45^, or down-regulated SREBPs degradation^46^.

In addition, elevated triglycerides as well as cholesterol concentration were observed in both SGA-treated rats, and these effects had a partly time-dependent character. However, a strong association between the increase in lipid levels and weight gain was not found, and this suggested the possibility of a direct effect on lipid levels by multiple mechanisms including hepatic SREBP as well as ChREBP pathways^26, 28^. In this study, OLZ and CLZ augmented the abundance of SREBP-1c and SREBP-2 in the nucleus without a significant effect on their precursor form in the cytoplasm. This suggests that they might have a strong effect in modulating the post-translational process of SREBP, which could trigger SREBP-1c/SREBP-2 activation and increase mRNAs for multiple lipogenic enzymes, thereby elevating lipid accumulation in the liver. Unexpectedly, a key player in lipogenesis, the nuclear ChREBP level was increased only in CLZ-treated rat livers. OLZ could not significantly activate nuclear ChREBP, possibly due to inhibition by OLZ-elevated NEFA. As an important regulatory factor in NEFA synthesis, ChREBP might have significantly lower expression levels in the liver with increased NEFA. ChREBP-β is a novel ChREBP isoform, and its mRNA is transcribed from a carbohydrate response element (ChoRE)-containing promoter located 17 kb upstream of the ChREBP-α (former ChREBP) transcriptional start site. Recently, it has been shown that ChREBP-β potently induces *de novo* lipogenesis, and its expression, regulated by glucose and by ChREBP-α in human adipose tissue and the liver, correlates with insulin sensitivity^47, 48^. Therefore, it will be interesting in future studies to analyse any possible change in ChREBP-β levels in the liver and WAT following OLZ and CLZ treatment.

Both CLZ and OLZ treatment are associated with the greatest risk of clinically significant weight gain in humans^49^, however, a number of studies have shown that only OLZ, but not CLZ, was observed to induce weight gain and enhance adiposity in rats. Rats treated with OLZ had higher fasting insulin, TG, and TC levels than controls, suggesting that OLZ should have a tendency to induce weight gain, dyslipidemia and hyperglycemia^5, 6, 50^. Our data demonstrated that the presence of fasting hyperinsulinemia which was associated with the body weight gain followed the time course of drug treatment, which could well contribute to insulin resistance and the enhancement of hepatic lipid levels. The increased insulin resistance in OLZ-treated rats was associated with reduced AKT activation in the liver, further contributing to high levels of circulating lipids and abdominal fat accumulation. Notably, CLZ was found to induce weight loss in this study, which did not mimic clinical findings but was consistent with previous reports in rats^19–21^. Lower body weight in CLZ-treated animals but without significant changes in WAT could mean that these animals had less lean mass. Although the mechanisms underlying the differences between OLZ and CLZ treatment on the body weight of rats is not clear, this study showed that the two drugs had different impacts on glucose metabolism, which may partially explain the different effects of CLZ and OLZ on body weight changes. Interestingly, our study uncovered a novel finding: following the time course of drug treatment, CLZ reduced insulin secretion which may be through a direct impairment of pancreatic β-cell function and increased fasting TC and TG levels (CLZ treatment at Week 1 induced significant body weight loss, so that CLZ seemed to have a lag phase in changing blood lipid levels compared to OLZ). This suggested that CLZ has direct effects on deficiency of insulin release and dyslipidemia. After long-term CLZ treatment, these metabolic disorders could further worsen the function of pancreatic β-cells, with failure to control blood glucose levels. Supporting our findings, several studies have reported that CLZ can directly act at rat β-cells to decrease insulin secretion^37, 38, 51^. Further studies testing insulin tolerance may provide more information for comparing the different effects of the two drugs. In addition, CLZ and OLZ might also have a direct effect on peripheral the lipogenic pathway, and cause lipid abnormalities, which appear to increase the risk of developing diabetes^52^.

In fact, these findings were parallel with clinical data that some patients with schizophrenia who did not experience any weight gain or who had lost weight suffered from diabetes and dyslipidemia^18, 49^. Collectively, SGAs are a heterogeneous class of drugs, each agent having differing pharmacological properties. These data suggested that weight gain or obesity might not be a single crucial factor for onset of metabolic side-effects, such as insulin resistance anddyslipidemia^42^. Notably, previous studies reported that OLZ and CLZ had a similar antagonist activity at histaminergic subtype 1 (H_1_) receptors, and the affinity for H_1_ receptors played a key role in drug-induced weight gain^53, 54^, however, metabolic side-effects could be associated with their plasma concentrations^55^. Further studies investigating a possible association between SGA serum concentrations and metabolic outcomes are needed.

In addition, the risk of hyperglycemia and dyslipidemia is correlated with drug affinity for certain mediator’s receptors such as histamine H_1_R and muscarinic M_3_R^10, 38, 56^. In this study, both OLZ and CLZ significantly promoted the expression of the two hepatic receptors by blocking them long term. In accordance with SGA-induced accumulation of lipid, we found increased hepatic H_1_R expression which was significantly positively correlated with elevated nuclear SREBP-1 levels as well as nuclear SREBP-2 levels in this study. Previously, it was shown that betahistine (a histamine H_1_R agonist) could act at H_1_R to ameliorate OLZ-induced dyslipidemia via the AMPKα-SREBP-1 and PPARα-dependent pathways in rats^56^. Therefore, as a potent histamine H_1_R antagonist, it is possible that during long-term OLZ/CLZ treatment, their antagonism at the hepatic H_1_R may contribute to an up-regulation of lipogenesis through the SREBPs signalling pathway. Furthermore, since the SGA blockade of M_3_R in pancreatic β-cells has been reported to reduce insulin release^37, 38, 57^, it would be important to use an M_3_R agonist to rescue the deficits of insulin secretion and/or other metabolic disorders caused by SGA blockade of M_3_R.

Female rats were used in this study, because the OLZ-induced weight gain model has been consistently established and validated in female rats in our and other laboratories^58–61^, while it could not be consistently modelled in male rodents^62^. Clinically, it is also a common observation that female patients have a much higher risk than males for OLZ-induced weight gain and other metabolic side-effects^63–66^.

**Figure 7:**
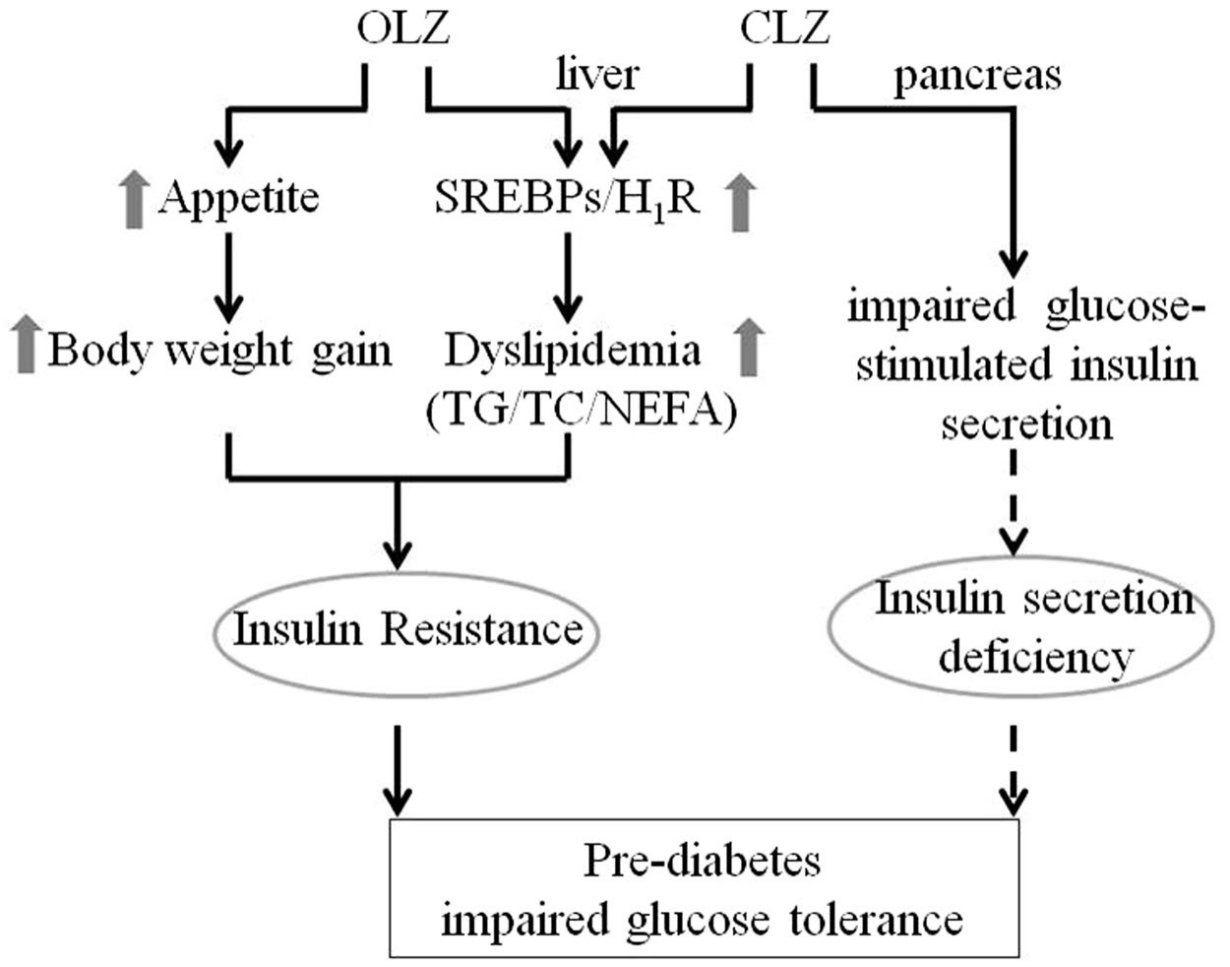
A schematic diagram of the proposed pathways for OLZ- and CLZ-induced glucose-lipid metabolic disorders.

In summary, the results of this study suggested a different pharmacological mechanism underlying the metabolic side-effects between OLZ and CLZ; a different time course could be responsible for impairment of glucose-stimulated insulin secretion, elevating fasting lipid levels, and developing insulin resistance in OLZ and CLZ treated rats. OLZ increased body weight gain and elevated hepatic lipid levels, and further induced insulin resistance which led to reduced fasting and postprandial glucose levels; CLZ had a strong direct role in lipid accumulation and insulin secretion deficiency, independent of weight gain, which could further induce serious impaired glucose tolerance (Fig. 7). Nevertheless, further studies are important to identify the molecular details in the regulation of abnormal lipogenesis and glucose metabolism associated with SGAs.

## Methods

### Animal studies

Thirty-six female Sprague-Dawley rats (200–220 g) were obtained from the Animal Resource Centre (Perth, WA, Australia). After one week of environmental familiarization, they were housed in individual cages and allowed *adlibitum* access to water and a standard laboratory chow diet (3.9 kcal/g; 10% fat, 74% carbohydrate and 16% protein) under environmentally controlled conditions (22 °C, with light cycle from 07:00–19:00 and dark cycle from 19:00–07:00) throughout the experimental period. Prior to drug treatment, rats were trained to take a sweet cookie dough pellet (0.3 g, including 30.9% cornstarch, 30.9% sucrose, 6.3% gelatin, 15.5% casein, 6.4% fibre, 8.4% minerals and 1.6% vitamins) without drugs twice daily (b.i.d.) for one week^59, 67^, and were randomly assigned into the following groups (n = 12/group) for nine-week treatment with OLZ (3 mg/kg, Zyprexa, Eli Lilly, Indianapolis, USA), CLZ (20 mg/kg, Clozaril, Novartis, Turkey), or control (vehicle, cookie dough mixed with water). The pellet with drug (0.3 g) was made prior to administration by mixing droplets of water with cookie dough powder. Each group received a twice-daily oral treatment (8:00 and 20:00 h, b.i.d.) of antipsychotic drug or vehicle for 9 weeks. Since the rats in the CLZ group avoided taking the cookie pellet with CLZ after 3 days’ treatment, CLZ was delivered via the mouth using a 1 ml syringe. To make sure the same amount of cookie pellets was taken in the three groups, the rats in the CLZ group continued to befed with sweet cookie dough pellets through the experimental period. Body weight, food intake and water intake of rats were measured once every week. All experimental procedures were approved by the Animal Ethics Committee, University of Wollongong, Australia (AE12/26), as well as complying with the Australian Code of Practice for the Care and Use of Animals for Scientific purposes (2004).

After completing treatment, all rats were sacrificed by carbon dioxide asphyxiation. Inguinal, perirenal and periovary white fat pads, as well as the liver, were dissected, individually weighed, and frozen in liquid nitrogen, followed immediately by storage in a −80 °C freezer until further analysis.

### Blood sampling and IGTT

At weeks 0, 1, 2, 3, 5, 7 and 9 of drug treatment, after overnight fasting, rats were anesthetized with isoflurane and held in a towel to minimize stress, while blood samples (0.2 ml) were collected into EDTA tubes byretro-orbital puncture. After centrifugation at 1000 g for 10 min at 4 °C, plasma was collected and stored at −80 °C prior to analysis. At Week 0 and Week 8, following overnight fasting, an IGTT was conducted (Fig. 5H), in which rats were administered an intraperitoneal injection of glucose (2 g/kg body weight, i.p). Underanesthesia with isoflurane, blood samples were collected by retro-orbital puncture immediately at 0 and 30 min after glucose injection for insulin and glucose measurements. Moreover, blood from the tail vein was analysed for glucose levels using an automatic glucometer (FreeStyle) at 0, 30, 60, 90, 120, and 180 min. The area under the curve (AUC) was calculated based on these measurements.

### Plasma glucose, insulin and lipid level measurements

Plasma insulin, TG and TC concentrations were measured using Thermo Scientific Kits on a Konelab 30i biochemistry analyser (Thermo Fisher Scientific Oy, Vantaa, Finland). Plasma insulin and glucose levels were measured using ELISA Kits (Merck Millipore, USA)^35^. Insulin resistance was calculated using the homeostasis model assessment (HOMA-IR: fasting glucose [mmol/L] x fasting insulin[mU/L]/22.5)^68^.

### Histological investigation

For the visualization of hepatic lipid content, cryostat sections were cut at 12 μm, fixed with 10% formalin for 5 minutes and lipid droplet deposition was detected by Oil-Red-O (ORO) staining (Sigma-Aldrich 01516, St Louis, MO, USA). Sections were rinsed with 60% isopropanol and stained for 15 min with filtered ORO solution (0.5% in isopropanol followed by 60% dilution in distilled water). After two rinses with 60% isopropanol and distilled water, slides were counterstained with hematoxylin (Sigma-Aldrich GHS232, St Louis, MO, USA) for 15 seconds, rinsed with water and mounted. For quantitative analyses of ORO staining, images were randomly sampled using a Leica DMRB microscope (Leica Systems, Toronto, Ontario, Canada) across 2 adjacent sections on the same slide (N = 6 individual livers) in each treatment group.The area of positive staining for ORO was calculated as a percentage of total section area, and an average lipid droplet size was calculated by utilizing morphometry software ImageJ (version 1.46)^35^.

### Isolation of total mRNA and analysis by qRT-PCR

Dissected liver was homogenized and RNA was isolated using RNA Mini Kit (Life Technologies, NSW, Australia) following the manufacturer’s instructions. RNA was converted to cDNA using cDNA Synthesis Kit (Life Technologies). RT-PCR was performed using LightCycler 480 Real-Time PCR instrument (Roche Applied Science, NSW, Australia) with the TaqMan Gene Expression Assays (Life Technologies): *Fasn* (Rn01463550 m1), *Acc1*(Rn00573474_ml), *Scd1* (Rn00821391_gl), *Lxr* (Rn00581185_m1), and *Hmgcr* (Rn00565598_m1). *β-actin* (Rn00667869_m1) and *gapdh* (Rn01775763_g1) were expressed as an endogenous control. The amplification was run for 40 cycles of denaturation at 95 °C followed by annealing/extending at 60 °C. All samples were analysed in duplicate. Results were expressed in relative expression using the comparative 2^−ΔΔCt^ method normalized by the housekeeping gene glyceraldehyde-3-phosphate dehydrogenase (*gapdh*) and *β-actin*in comparison to controls. The mean value of the control group was set at 1 and all data were normalized versus the control group.

### Preparation of nuclear and cytoplasmic extracts, and Western blot analysis

Nuclear protein lysates of liver tissue were prepared from rat livers using the nuclear extraction reagent kit (Pierce Biotechnology, USA), according to the manufacturer’s instructions. Whole-protein lysates of liver tissue were extracted using 10% Nonidet P-40 lysis buffer (Invitrogen, Camarillo, CA, USA) supplemented with 1% protease inhibitor cocktail (Sigma-Aldrich), 0.5 mM β-Glycerophosphate (Invitrogen) and 1.0 mM phenylmethylsulfonyl fluoride (Sigma-Aldrich). Protein concentration was detected by SpectraMax Plus 384 absorbance microplate reader (Molecular Devices, USA) using the Bio-Rad Assay. Aliquots containing 10 μg of proteins were loaded onto an 8% to 12% sodium dodecyl sulfate–polyacrylamide gel, transblotted onto polyvinylidene difluoride (PVDF) membrane (Bio-Rad), blocked with 5% BSA in Tris-buffered saline with 0.1% Tween-20, and then incubated with the primary antibodies including anti-SREBP-1 (1:1000, Santa Cruz, sc-364), anti-SREBP-2 (1:1000, Abcom, ab30682), anti-ChREBP (1:2000, Santa Cruz, sc-33764), anti-histamineH_1_R (1:1000, Santa Cruz, # SC-20633), anti-M_3_R (1:1000, Santa Cruz, sc-9108), anti-AKT (1:2000, Cell Signaling, #4691), anti-phospho-AKT (Ser473) (1:1000, Cell Signaling, #4060), anti-GSK3β (1:2000, Cell Signaling, #5676), anti-phospho-GSK3β (1:1000, Cell Signaling, #9322) and anti-β-actin (1:2000, Santa Cruz, sc-47778). The membrane was then incubated with horseradish peroxidase–conjugated goat anti-rabbit (1:5000, Millipore) or goat anti-mouse IgG (1:5000, Millipore). The bound complexes were detected with Amersham Hyperfilm ECL (GE Healthcare, Life Science, USA) and quantified by a GS-800 image densitometry (Bio-Rad). The ratio to β-Actin was calculated and presented as fold changes, setting the values of control rats as one.

### Statistical analysis

Data are presented as mean ± standard error. The Kolmogorov-Smirnov test was used to examine the distribution of data from all experiments. Weight gain, food intake, fasting glucose, insulin, TG, and TC levels were analysed by two-way repeated ANOVAs (Treatment × Time as repeated measures), followed by the post-hoc Dunnett-T test for multiple comparisons. One-way ANOVA was used to analyse gene expression and protein level data. For the data without a normal distribution, data were analysed using the Kruskal–Wallis H test, followed by a post-hoc Mann-Whitney U test at each time point. Pearson’s or Spearman correlation tests were used to assess the relationships among these measurements. All data were presented as mean ± SEM, and statistical significance was accepted when *p* < 0.05.

## Electronic supplementary material

Supplementary information
