# Supplementary material for: Time-dependent changes and potential mechanisms of glucose-lipid metabolic disorders associated with chronic clozapine or olanzapine treatment in rats
Source: Sci Rep. 2017 Jun 5;7:2762. doi: 10.1038/s41598-017-02884-w (PMC5459828; doi:10.1038/s41598-017-02884-w)
Supplement: Supplementary file 1 — Supplementary information [file 41598_2017_2884_MOESM1_ESM.pdf]

Title: Time-dependent changes and potential mechanisms of glucose-lipid metabolic disorders associated with chronic clozapine or olanzapine treatment in rats

Authors: Xuemei Liu[1,2,3], Zhixiang Wu[2], Jiamei Lian[2,3], Chang-Hua Hu[1], Xu-Feng Huang[2,3], Chao Deng[2,3*]

1: School of Pharmaceutical Sciences, Southwest University, Chongqing 400715, PR China

2: Illawarra Health and Medical Research Institute, Wollongong, 2522, NSW, Australia

3: School of Medicine, University of Wollongong, Wollongong, 2522, NSW, Australia

***Corresponding authors:**

Professor Chao Deng, Antipsychotic Research Laboratory, IHMRI, University of Wollongong, NSW, 2522, Australia. Tel.: +61 2 4221 4934, Fax: +61 2 4221 8130. E-mail: chao@uow.edu.au

Figure 2

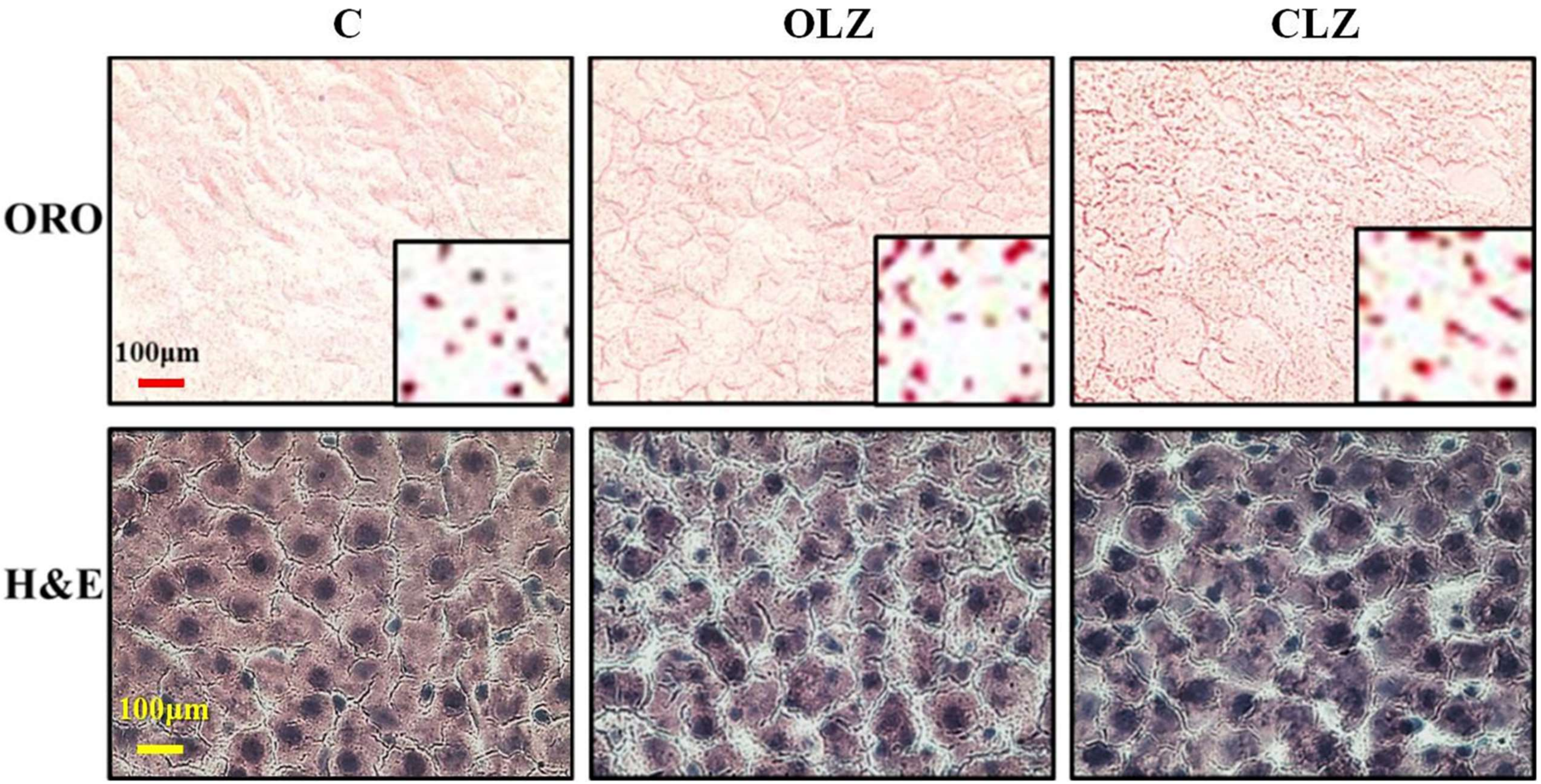

Figure 3

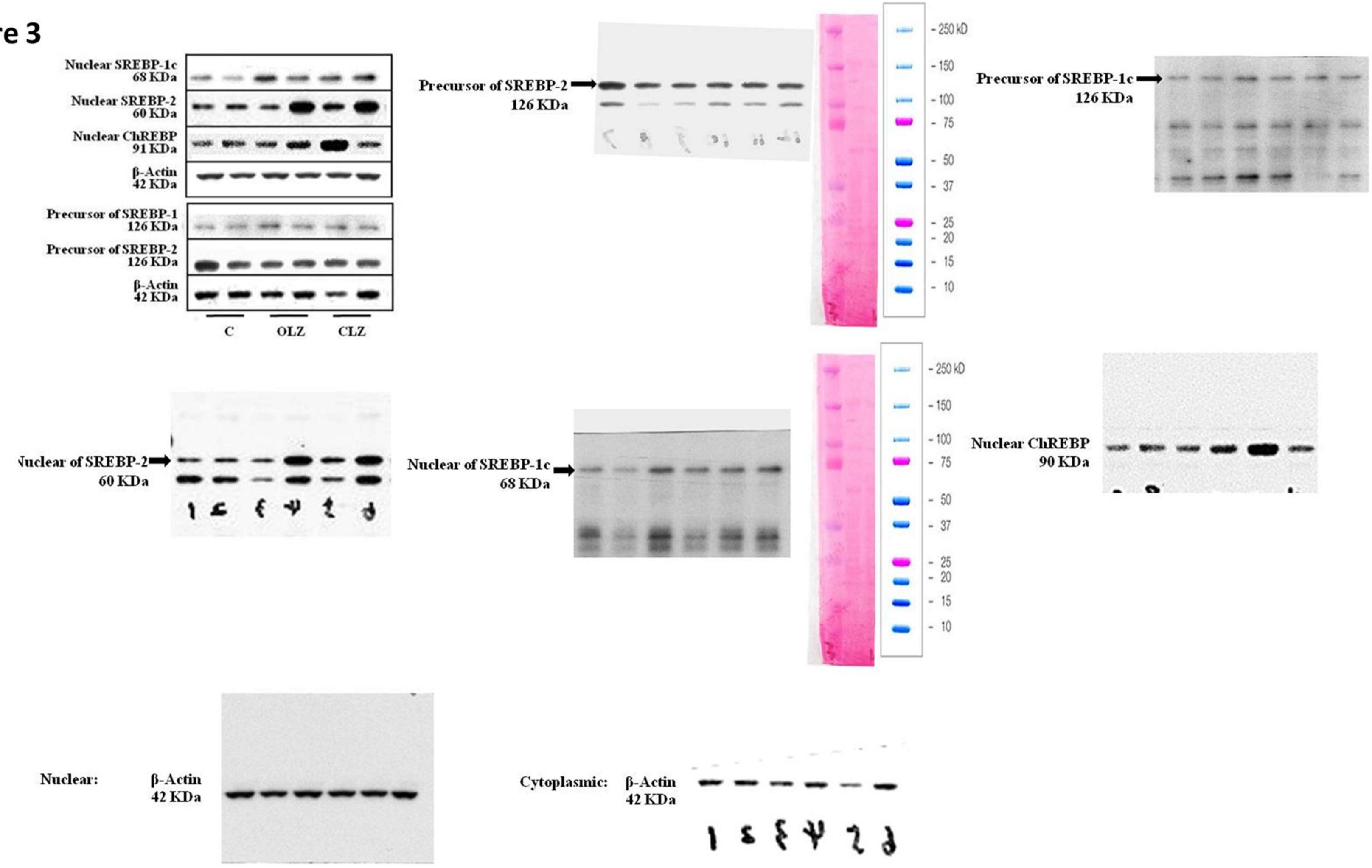
Nuclear SREBP-1c 68 KDa
Nuclear SREBP-2 60 KDa
Nuclear ChREBP 91 KDa
β-Actin 42 KDa
Precursor of SREBP-1 126 KDa
Precursor of SREBP-2 126 KDa
β-Actin 42 KDa
C
OLZ
CLZ
Precursor of SREBP-2 126 KDa
250 kD
150
100
75
50
37
25
20
15
10
Precursor of SREBP-1c 126 KDa
Nuclear of SREBP-2 60 KDa
Nuclear of SREBP-1c 68 KDa
250 kD
150
100
75
50
37
25
20
15
10
Nuclear ChREBP 90 KDa
Nuclear:
β-Actin 42 KDa
Cytoplasmic:
β-Actin 42 KDa

Figure 5

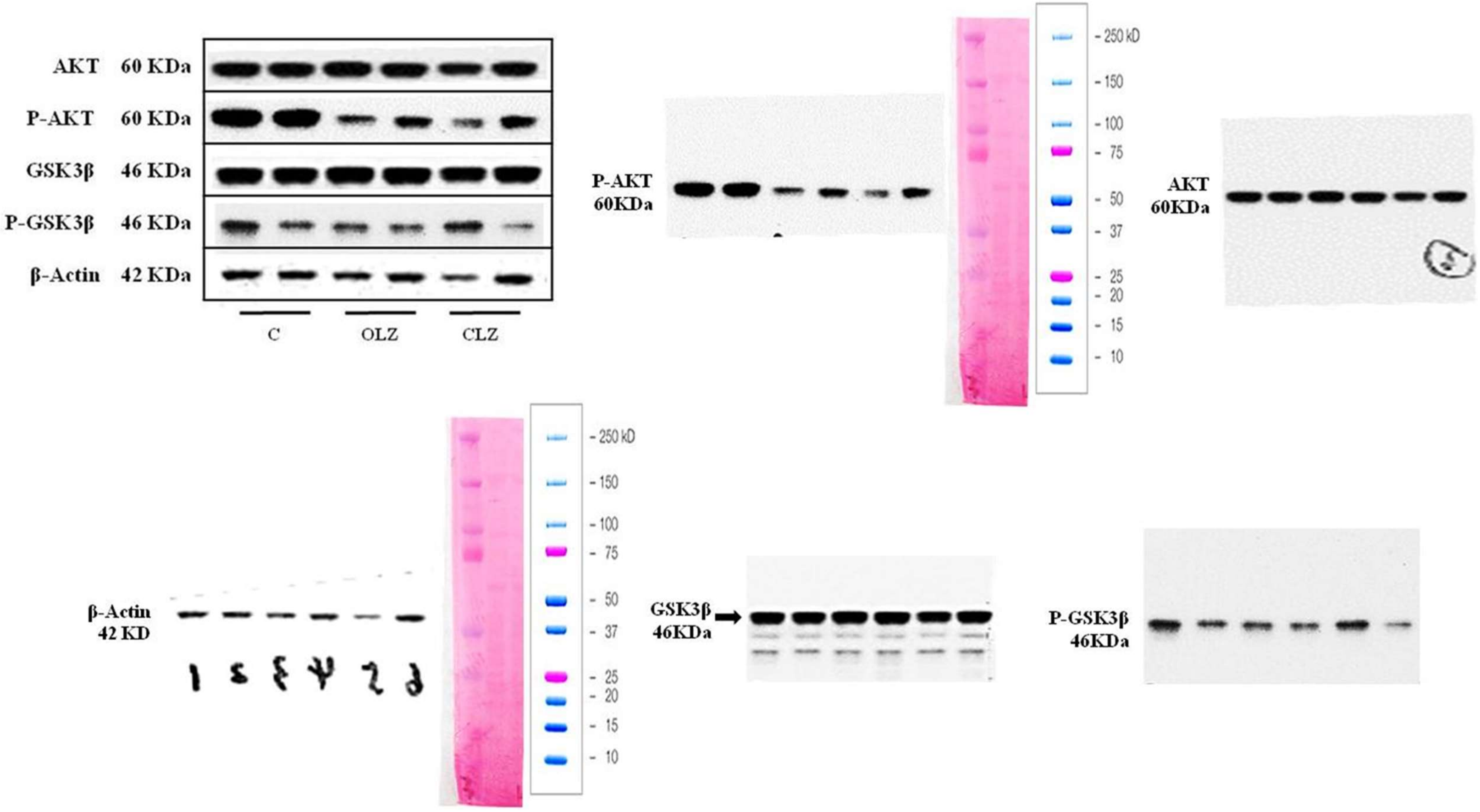
AKT 60 KDa
P-AKT 60 KDa
GSK3β 46 KDa
P-GSK3β 46 KDa
β-Actin 42 KDa
C
OLZ
CLZ
P-AKT
60KDa
250 kD
150
100
75
50
37
25
20
15
10
AKT
60KDa
β-Actin
42 KD
250 kD
150
100
75
50
37
25
20
15
10
GSK3β
46KDa
P-GSK3β
46KDa

Figure 6

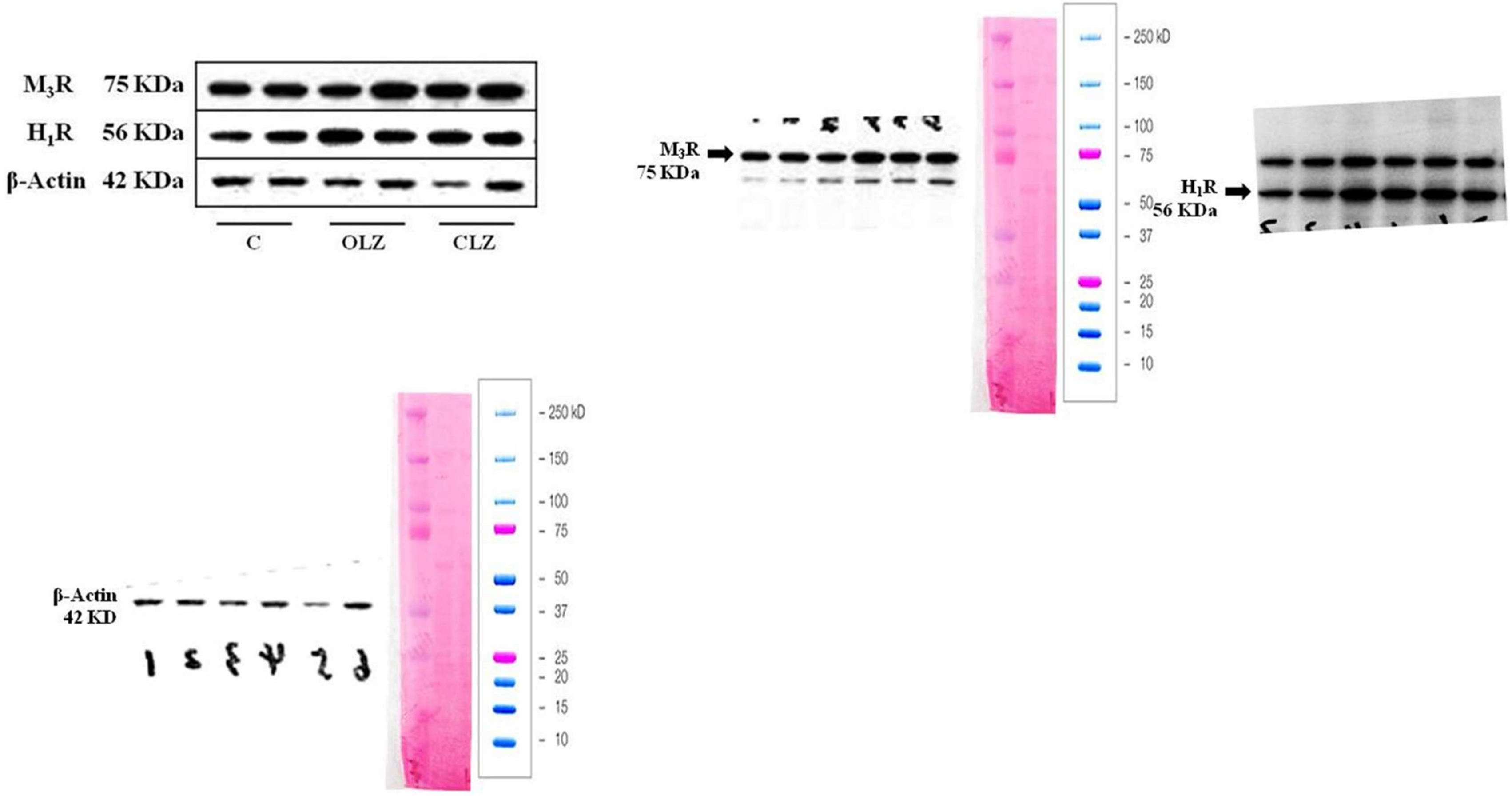

M3R 75 KDa
H1R 56 KDa
β-Actin 42 KDa
C
OLZ
CLZ
M3R
75 KDa
250 kD
150
100
75
50
37
25
20
15
10
H1R
56 KDa
β-Actin
42 KD
250 kD
150
100
75
50
37
25
20
15
10
